# Mr. Hume's Remarks on His Arsenical Test

**Published:** 1812-10

**Authors:** Joseph Hume

**Affiliations:** Long Acre, London


*To the Editors of the Medical and Physical Journal.*

GENTLEMEN,

NOW, that my letters on the *test for arsenic*, together with what Dr. Henry has published upon the same subject, have been attentively read by Dr. Roget, I am astonished to find, by this gentleman's letter to you in the last Number of your Journal, that he still perseveres in the same errors, particularly on two points; that of perverting the text of my letters, and of ascribing *any* part of the discovery of a test by *silver* to Dr. Marcet.

Through the whole of Dr. Roget's "reply," there is not the slightest allusion to the *occasion* which called for my two letters in your Journal. The first letter contained some strictures upon a specific case, and upon the methods which had been pursued by your correspondent, Mr. Jones, of Howden, with a view to detect arsenic. It is the case of Ann White, in the Journal of April 1810, in which it is stated, that, from the stomach of this unfortunate victim to the poison, a considerable quantity of *a white powder suspected to be arsenic* had been collected; and enough, I should suppose, to make a great number of analyses, even on the *old* plans heretofore in use.

It will be seen, by my second letter, that, from motives of delicacy, I did not continue my animadversions on Mr. Jones's efforts; I did not say even that *nine* grains of subcarbonate of potash were too much for *one* grain of the *suspected white powder;* nor, for the same reasons, did I accept of Mr. Jones's polite offer of sending me, for an analysis, the five grains of the powder remaining in his possession. We are strangers to each other, and therefore it would have been unbecoming in me, and it was contrary to my feelings, to have pushed the question beyond my second letter, to which I purposely affixed the title of "*Cursory Remarks on Arsenical Preparations.*"

I will now point out where I think Dr. Roget has perverted the text; and this he has done, not only in his "case of recovery," as published in the Medico-Chirurgical Transactions, but it is repeated, with aggravated circumstances, in his "reply," in which he attempts to prove the former assertion.

The very objectionable note in Dr. Roget's case goes to state, that "Mr. Hume has proposed *boiling* the suspected matter *with a solution of carbonate of potash*, and bringing into contact with it a stick of dry nitrate of silver; a method of proceeding analogous to the one I (Dr. R.) have described, but which appears less convenient in its practical application."

Now, in my first letter to you, I advised Mr. Jones to boil "*three grains* of the suspected powder in not less than eight ounces ounces of water." Here there is an exact prescription adapted to a particular case; not a word about potash, soda, lime, or any other alkali, till this same powder *has been boiled.* Then it is directed that a *single grain or two* of subcarbonate of potash or soda is to be *added*, agitating the vessel to make the mixture uniform; and here there follows a caution, which will prove that the suspected matter is positively forbidden to be *boiled with* the alkali. "By *adding* the alkali *after* the solution has boiled, we are enabled to decide whether any part, or the whole, of the suspected powder has been dissolved, &c."

Dr. Roget must not suppose me to be such a novice in my profession as to employ the operation of *boiling* in *all* cases; that I should make no difference between this dry powder and any suspected *fluid*, where there is no sediment, such as tea, coffee, wine, or water; nor that the alkaline solution is to be added at random, and without limitation in its quantity, as your readers will infer from his quotation.

That Dr. Roget had never tried my test with the *fixed* alkalies, nor with the alkaline earths, I have a right to infer, from the character he has thought proper to bestow upon it by the expressions,—"analogous" to the supposed new test, "but which *appears* less convenient in its *practical* application." I consider it, therefore, highly ungenerous to depretiate the labors of any one without some decided proof, by experiments fairly described, rather than by the mere assertion, that my test *appears* to be inferior to another, which by the way also originated from myself. It is more usual to say that the son is *like to his father*, than the contrary; and Dr. Roget should have expressed himself in the same style; "that the test, when conducted by ammonia, is analogous to *all* the varieties either prescribed as formulæ in Mr. Hume's letters, or included under his own words *soda*, *potass*, *lime*, or *any other alkali*, provided SILVER be employed." This would have been candid, and no more than the truth.

There is a passage in the "reply" which proves that Dr. Roget may yet gain more knowledge in chemistry by experience. It is this: "That the employment of ammonia is much more appropriate than any of the alkalies or earths which Mr. Hume has pointed out, is, I think, pretty evident, from its not requiring *previous* boiling *with* the suspected matter, and still more from its occasioning *no precipitate with nitrate of silver alone*, as the fixed alkalies do; a circumstance which, in *unskilful* hands, would be exceedingly apt to lead to equivocal results."

The best answer I can give to all this, is, to transcribe the method which I should pursue, when in search of the oxide of arsenic by the medium of ammonia, being the same which I have published in the Philosophical Magazine for last month, month, and which will prove that there *is* a precipitate formed, when ammonia is added to nitrate of silver. I am the more inclined to take this step, presuming that it may be very acceptable to such of your numerous readers who may not read that valuable Journal, to be informed of the most ready process to prepare my test, under the title of

"*Ammoniaco-nitrate of Silver.*"

"Dissolve a few grains, say ten, of the fused nitrate of silver commonly called lunar caustic, in about nine or ten times its weight of distilled water; to this add, by a drop at a time, some liquid ammonia till a *precipitate* be formed. Continue cautiously to add the ammonia, now and then shaking the bottle, till the precipitate shall be taken up and the solution again become transparent, or nearly so, as the ammonia need not be in great excess; if in any; for, solution of ammonia being lighter than water, the superfluous portion would be likely to remain on the surface of the fluid, to which this test-liquor is to be applied,—a circumstance not noticed by other analysts."

My criticisms upon what Dr. Roget has said, respecting Fowler's arsenical solution, are perfectly correct, and in no wise weakened or invalidated by any thing contained in his "reply;" since it will be no difficult matter to prove that, in the experiment which these gentlemen made upon this solution, the test was positively reduced to the precise formula I had before projected; and that the *nitric acid* in this case assuredly arranged itself towards the *potash*, the ammonia remaining nugatory as a superincumbent stratum; while the *oxide of silver*, on the other hand, is attracted by the arsenic, or rather the *arsenious acid*. The white oxide of arsenic is, like some other compounds of oxygen, to be considered as an acid *only when combined with water;* such, however, is my conception of the nature of this acidifiable base.

But let us prove the nullity of the ammonia, in its application as a test to *Fowler's* arsenical solution, by a fair experiment.

Take two small phials, each capable of holding about two ounces of distilled water; fill them nearly with this water, and add to each about twenty drops of Fowler's solution, shaking the phials to mix the fluids. Into one phial pour with caution as much liquid ammonia as will form a visible stratum upon the surface of the mixture; and then present the fused nitrate of silver, either in the dry state, or in solution, to the surface of each bottle of fluid. Notwithstanding this *surplus* of ammonia in *one* phial, the *silver* will make its way to the *arsenic*, and the *nitric acid* to the *potass*, in *both* the experiments.

On agitating the phial which contains the ammonia, the yellow precipitate will, I know, disappear, and the liquor again become transparent. To this, however, I should not object, provided provided I have been satisfied of the *presence of arsenic;* this being our first object and the sole purpose of such a research. The inference I should draw from all similar experiments, is, that where potass, soda, or any other body is present, that has a greater affinity for nitric acid than *ammonia*, this last alkali may as well be omitted, being quite superfluous.

It appears, by Dr. Roget's "case of recovery, &c." that the patient had taken, but a *few hours* before his first visit, three large doses of potass. The mixture contained *nearly* one ounce of the subcarbonate of potass in eight ounces of the peppermint water; and, as a small part of the alkali would pass to the sulphuric acid, there would necessarily be also carbonate of magnesia in the same medicine. It is likely, therefore, that more than sixty grains of the carbonates of potass and magnesia together were contained in each dose; making 180 grains at least of alkaline matter in the three doses, administered in the course of a few hours before Dr. Roget first saw the patient on the 13th of February.

I make no doubt that this very material fact did not pass unobserved by Dr. Roget, and his coadjutor Dr. Marcet; but, as it does not appear that the fluid rejected from the patient's stomach had been corrected by the addition of acid, if it required it, in order to fit it for the proper tests; nor, do I find, by Dr. Roget's silence on this head, that any previous experiments were made to decide whether an excess of alkali with carbonate of magnesia might not be present in this fluid; I must, consequently, hesitate before I subscribe myself to be fully satisfied with the accuracy of their mode of operating,—at least with nitrate of silver.

This omission of Dr. Roget may possibly explain the following passage in the "case," which, I confess, I do not otherwise comprehend. "The fixed alkalies, when substituted for ammonia, likewise produce a yellow precipitate; but the results are less distinct, since, *in the circumstances in which the experiment is made*, they decompose the nitrate of silver, an effect which ammonia does not produce."

Every unprejudiced person, speaking of the arsenical test, would consider its excellence to depend solely upon the *silver*, provided the experiments be conducted according to the principles of double elective attraction; the alkali, earth, or other base that may be employed, of which there is a greater choice than is generally allowed, being a secondary consideration. Thus, as a test for sulphuric acid, we name *barytes;* for iron, *prussic acid* or *gallic acid;* for lime, *oxalic acid;* for silver, *muriatic acid:* and in this way we distinguish every test whatever, many of which act reciprocally, particularly my own; for, as silver is a test for *arsenic*, so may arsenic become a test for *silver*.

It

It is by this rule that Dr. Henry has so properly and clearly defined my test, in his "Elements of Experimental Chemistry." Such high authority ought to have been considered by Dr. Roget as a very sufficient reason for renouncing all claim, in favor of his friend's pretensions as a discoverer; at least as far as the question of arsenical test with *silver* is concerned.

Though Dr. Henry quotes my letter in the Philosophical Magazine, as well as those in your Journal, and, consequently had the choice of *soda, lime, potash*, or *any other alkali* or *alkaline earth*, from which he might have derived a name for the test, to distinguish it from others; he refused all these, and made his election, like a man of liberality and real science, by fixing on the *silver*. Dr. Henry's words are these:

"A new process for detecting arsenic has been proposed by Mr. Hume, of London, in the Philosophical Magazine for May 1809, vol. xxxiii. The *test* which he has suggested is the *fused nitrate of silver* or lunar caustic, which he employs in the following manner."

Dr. Henry then proceeds to detail *one* of the examples I have prescribed, though not altogether in my own words; namely, the process in your Journal for June 1810, being the same I offered, in a particular case, to your correspondent Mr. Jones, by whom it was acknowledged in the next Number of your pages, the Number for July.

So far, therefore, as this question is connected with Dr. Henry's name, I have nothing to fear, nothing to regret; that gentleman has assumed no merit to himself, nor has he endeavored to influence his readers; and, on my side, I beg to own myself much indebted to him for having included this test in his work.

I am sorry, however, to find that the test had not been under the practical experience of this very able master of the science, before he wrote his "Elements of Experimental Chemistry;" otherwise I am satisfied the process of reducing the arsenic to the metallic state, as proposed by Dr. Black,* would have been ranked as an inferior and a mere secondary auxiliary, when compared to mine. Thus, the *single grain* of the white oxide of arsenic, which is required for *one* experiment in this way, would, under my direction, serve amply for more than *one thousand.* In a future edition of this meritorious work, its eminent author will, no doubt, prefer the silver test to all others, and place it at the top of his list, where it will probably always remain.

I shall now, gentlemen, commit this question, respecting the difference of opinion between Dr. Roget and myself, to

* Henry's "Elements of Experiment. Chem." vol. ii. p. 391.

your your decision, and that of your chemical readers; trusting that, by thus making a public appeal, Dr. Roget, as well as Dr. Marcet and myself, shall be mutually satisfied with the consequent award.

The question seems capable of being reduced merely to this; What is comprehended under such expressions as *potass*, *lime*, *soda*, or *any other alkali?* Does not this language include *ammonia*, *barytes*, *strontites* and *magnesia* at least?

One thing is evident, and it must influence every one who will candidly consider this question: it is this,—that Dr. Marcet could *not* proceed without my *nitrate of silver;* while I, on the contrary, can readily forego *any one* of the alkalies or alkaline earths, provided I be allowed to make my choice among those which remain: therefore, under the strongest assurance of security, and a conviction that my claim is irresistibly fixed, I shall abstain from making further remarks, unless I be compelled to resume the subject.

I remain, Gentlemen,

*Long Acre, London,* Your obliged and obedient Servant,

*August* 9, 1812. JOSEPH HUME.

